# Inter-reader agreement of the PI-QUAL score for prostate MRI quality in the NeuroSAFE PROOF trial

**DOI:** 10.1007/s00330-021-08169-1

**Published:** 2021-07-29

**Authors:** Francesco Giganti, Eoin Dinneen, Veeru Kasivisvanathan, Aiman Haider, Alex Freeman, Alex Kirkham, Shonit Punwani, Mark Emberton, Greg Shaw, Caroline M. Moore, Clare Allen

**Affiliations:** 1 Department of Radiology, University College London Hospital NHS Foundation Trust, London, UK; 2 Division of Surgery & Interventional Science, University College London, 3rd Floor, Charles Bell House, 43-45 Foley St, London, W1W 7TS UK; 3 Department of Urology, University College London Hospital NHS Foundation Trust, London, UK; 4 Department of Pathology, University College London Hospital NHS Foundation Trust, London, UK; 5 Centre for Medical Imaging, University College London, London, UK

**Keywords:** Urogenital neoplasms, Prostatic neoplasms, Magnetic resonance imaging

## Abstract

**Objectives:**

The Prostate Imaging Quality (PI-QUAL) score assesses the quality of multiparametric MRI (mpMRI). A score of 1 means all sequences are below the minimum standard of diagnostic quality, 3 implies that the scan is of sufficient diagnostic quality, and 5 means that all three sequences are of optimal diagnostic quality. We investigated the inter-reader reproducibility of the PI-QUAL score in patients enrolled in the NeuroSAFE PROOF trial.

**Methods:**

We analysed the scans of 103 patients on different MR systems and vendors from 12 different hospitals. Two dedicated radiologists highly experienced in prostate mpMRI independently assessed the PI-QUAL score for each scan. Interobserver agreement was assessed using Cohen’s kappa with standard quadratic weighting (κw) and percent agreement.

**Results:**

The agreement for each single PI-QUAL score was strong (κw = 0.85 and percent agreement = 84%). A similar agreement (κw = 0.82 and percent agreement = 84%) was observed when the scans were clustered into three groups (PI-QUAL 1–2 vs PI-QUAL 3 vs PI-QUAL 4–5). The agreement in terms of diagnostic quality for each single sequence was highest for T2-weighted imaging (92/103 scans; 89%), followed by dynamic contrast-enhanced sequences (91/103; 88%) and diffusion-weighted imaging (80/103; 78%).

**Conclusion:**

We observed strong reproducibility in the assessment of PI-QUAL between two radiologists with high expertise in prostate mpMRI. At present, PI-QUAL offers clinicians the only available tool for evaluating and reporting the quality of prostate mpMRI in a systematic manner but further refinements of this scoring system are warranted.

**Key Points:**

• *Inter-reader agreement for each single Prostate Imaging Quality (PI-QUAL) score (i.e., PI-QUAL 1 to PI-QUAL 5) was strong, with weighted kappa = 0.85 (95% confidence intervals: 0.51 – 1) and percent agreement = 84%.*

• * Interobserver agreement was strong when the scans were clustered into three groups according to the ability (or not) to rule in and to rule out clinically significant prostate cancer (i.e., PI-QUAL 1-2 vs PI-QUAL 3 vs PI-QUAL 4–5), with weighted kappa = 0.82 (95% confidence intervals: 0.68 – 0.96) and percent agreement = 84%.*

• *T2-weighted acquisitions were the most compliant with the Prostate Imaging Reporting and Data System (PI-RADS) v. 2.0 technical recommendations and were the sequences of highest diagnostic quality for both readers in 95/103 (92%) scans, followed by dynamic contrast enhanced acquisition with 81/103 (79%) scans and lastly by diffusion-weighted imaging with 79/103 (77%) scans.*

**Supplementary Information:**

The online version contains supplementary material available at 10.1007/s00330-021-08169-1.

## Introduction

The development and subsequent diffusion of multiparametric magnetic resonance imaging (mpMRI) of the prostate have unavoidably resulted into variability in terms of vendors’ and scanners’ quality worldwide [1].

Suboptimal image acquisition reduces the diagnostic accuracy of mpMRI for the detection of clinically significant prostate cancer and this is why since the publication of the first version, the Prostate Imaging Reporting and Data System (PI-RADS) committee outlined the technical requirements for the acquisition of mpMRI of the prostate of adequate diagnostic quality [2].

Moreover, two boards of experts have reiterated the importance of quality criteria for the acquisition of mpMRI of the prostate [3,4], and the Prostate Imaging Quality (PI-QUAL) scoring system [5] from the multi-centre PRECISION trial [6] has represented the first attempt to address this issue.

The PI-QUAL score evaluates the quality of prostate mpMRI against objective technical criteria (as per PI-RADS guidelines) together with subjective criteria from the images [5].

PI-QUAL is centred on a 1-to-5 scale that assesses the adequacy of the diagnostic quality of mpMRI of the prostate, where 1 implies that all mpMRI sequences are below the minimum standard of diagnostic quality, 3 means that the study is of sufficient diagnostic quality (as at least two mpMRI sequences taken together are of diagnostic quality), and 5 indicates that all sequences are of optimal diagnostic quality. In more detail, a PI-QUAL score ≥ 4 implies that the quality of the study is high (i.e., all clinically significant lesions can be ruled in and ruled out).

The purpose of this study was to evaluate the inter-observer agreement of the PI-QUAL score in patients enrolled in the NeuroSAFE PROOF trial [7].

## Materials and methods

This is a retrospective analysis of men prospectively enrolled in the NeuroSAFE PROOF trial (registration number: NCT03317990), whose recruitment started in 2018 [7]. The trial received ethical approval (Regional Ethics Committee reference 17/LO/1978) and was supported by the National Institute for Healthcare Research (NIHR reference PB-PG-1216-20013). Written informed consent was obtained from all patients, who granted permission for their samples to be used for research purposes. No further ethical approval was needed for this specific audit.

The NeuroSAFE PROOF trial is an ongoing multicentre randomised controlled trial in which patients are randomised 1:1 to either NeuroSAFE (a technique that involves intraoperative fresh-frozen section analysis of the posterolateral aspect of the prostate margin to assess whether cancer extends beyond the capsule) or standard robot-assisted radical prostatectomy [7].

### Selection of patients

Participants were recruited from different cancer centres routinely performing at least 250 cases of radical prostatectomy per year.

According to local protocols, all patients received a biparametric or multiparametric MRI study of the prostate prior to surgery on different MRI systems (Philips®, Siemens®, or General Electric®), on either a 1.5- or 3-T scanner, all without endorectal coil. During the trial, all scans were reported by dedicated genitourinary radiologists at each participating centre with at least 2 years of experience in prostate MRI reporting. At the same time, all scans were stored in a dedicated secured repository from which two designated radiologists (F.G. and C.A.) from the coordinating centre were able to download the scans for the assessment of image quality. As the PI-QUAL score [5] implies mpMRI of the prostate (i.e., including T2-weighted imaging (T2-WI), diffusion-weighted imaging (DWI) and dynamic contrast-enhanced (DCE) sequences), we excluded those patients who received a biparametric scan.

### Assessment of image quality

**Fig. 1:**
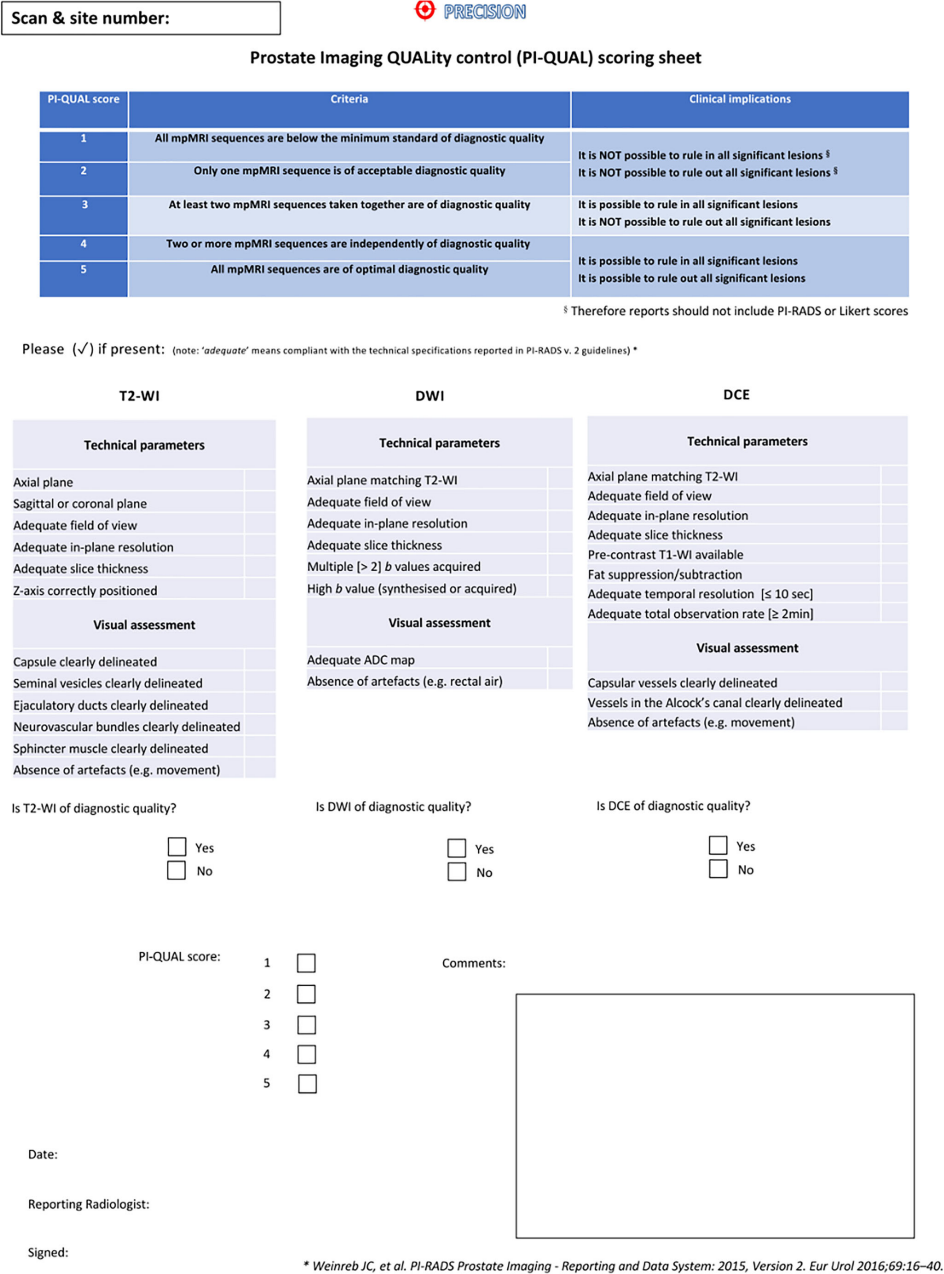
The PI-QUAL scoring sheet used to assess the quality of multiparametric magnetic resonance imaging. Legend: T2-WI, T2-weighted imaging; DWI, diffusion-weighted imaging; DCE, dynamic contrast–enhanced; ADC, apparent diffusion coefficient. Reprinted with permission from Giganti et al. [5]

Two radiologists highly experienced in prostate MRI reporting (F.G., a fellowship-trained consultant radiologist reporting around 2000 prostate MRI scans per year and with 7 years of experience in prostate MRI, and CA, a senior consultant radiologist reporting more than 3000 prostate MRI scans per year and with 20 years of experience in prostate MRI) evaluated the image quality independently using the published PI-QUAL scoring sheet (Fig. 1). The adherence to technical parameters was tested against the PI-RADS v. 2.0 guidelines (as trial recruitment started in 2018, before the publication of the PI-RADS v.2.1 guidelines) [8,9], as also stated in the original publication [5].

In detail, both readers assessed technical parameters such as field of view, in-plane resolution, and slice thickness for all MRI sequences and other specific parameters related to each single sequence (e.g. temporal resolution for DCE) and they visually assessed anatomical structures (e.g. the prostatic capsule on T2-WI or the pudendal vessels in the Alcock’s — or pudendal — canal on DCE) using a dedicated Picture Archiving and Communication System (PACS) viewer (Vue PACS, Carestream Health, Inc).

Both readers were blinded to all clinical (including the original report and site key) and pathological information. All scans were anonymised and were read in randomised order by each reader.

It should be noted that although the two radiologists were blinded to the original report, for the purposes of this specific study they did not re-report the scans (i.e., no lesions were scored) but they deliberately focussed their work on the sole assessment of image quality.

### Pathologic analysis

All patients received radical prostatectomy, either standard or using NeuroSAFE, at a single regional academic uro-oncology unit participating in the NeuroSAFE PROOF randomised controlled trial. The centre receives referrals from multiple centres in the vicinity as part of the National Health Service hub and spoke model for the referral of prostate cancer for surgery [7]. All pathological specimens were reported by dedicated urogenital pathologists.

### Statistical analysis

Clinical and demographic data are reported using descriptive statistics. Continuous variables are summarised by median and interquartile ranges and categorical data by frequencies and percentages.

Inter-observer agreement was calculated using two methods: the percent agreement (defined as the total number of concordant readings divided by the total number of readings made) and Cohen’s kappa with standard quadratic weighting (κw) using the formula: *ω*_*i*_ = 1− $\frac{i^2}{{\left(k-1\right)}^2}$ , where *i* is the difference between categories and *k* is the total number of categories. Cohen’s kappa coefficients (ranging from 0 to 1) were interpreted as follows: 0.01–020, slight agreement; 0.21–0.40, minimal agreement; 0.41–0.60, moderate agreement; 0.61–0.80, substantial agreement; 0.81–0.90, strong agreement; and >  0.90, almost perfect agreement [10–12].

Statistical analyses were performed using SPSS (IBM, version 27).

## Results

**Fig. 2:**
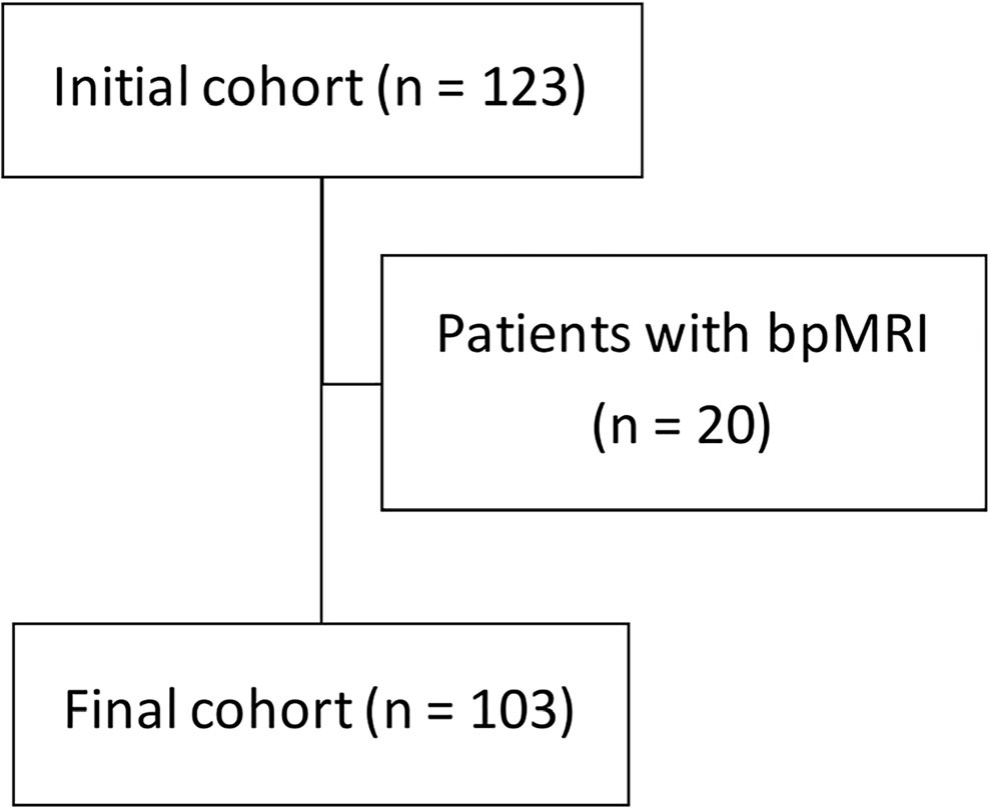
Flowchart of the cohort included in this study. Legend: bpMRI, biparametric magnetic resonance imaging

Among 123 patients from 13 centres who were enrolled in the study (as of February 2021), 20 (all from the same centre) had biparametric MRI, leaving 103 patients from 12 centres for final analysis (Fig. 2).

Patient characteristics are shown in Table 1.

**Table 1:** Characteristics of patients included in the study

	Population (n = 103)
Age (years)	57 [52–61]
PSA (ng/ml)	6.9 [5.1–9.4]
Prostate volume (cc)	34 [26–41]
PSA density (ng/ml/ml)	0.21 [0.15–0.32]
Gleason Grade group at biopsy	
Gleason Grade 1 Gleason Grade 2 Gleason Grade 3 Gleason Grade 4	4 (4%) 85 (82%) 9 (9%) 5 (5%)
Gleason Grade group at radical prostatectomy	
Gleason Grade 1 Gleason Grade 2 Gleason Grade 3 Gleason Grade 4 Gleason Grade 5	1 (1%) 84 (81%) 16 (16%) 0 2 (2%)
Pathological T stage at radical prostatectomy	
T2a T2b T2c T3a T3b	3 (3%) 3 (3%) 66 (64%) 26 (25%) 5 (5%)

Data are medians with interquartile ranges in brackets or number of patients with percentages in parentheses

**Fig. 3:**
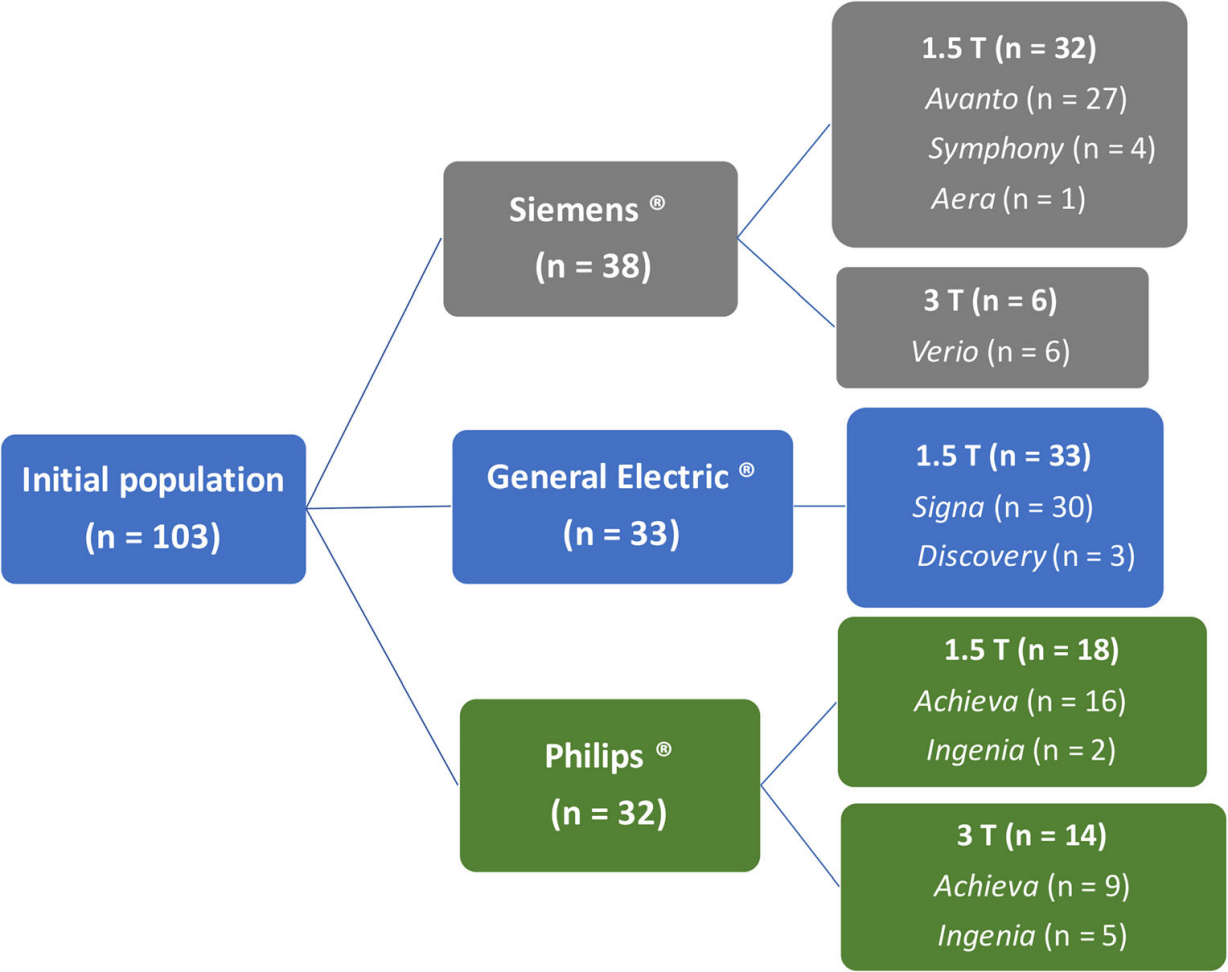
MR manufacturers and systems included in the study

Overall, 83/103 (81%) patients were scanned on a 1.5-T scanner and 20/103 (19%) patients on a 3-T scanner, and the distribution in terms of MRI systems and manufacturers is shown in Fig. 3. The distribution of scans for each participating centre is reported in Table 2.

**Table 2:** Distribution of MR scans for each participating centre

	Number of MR scans
Centre 1	31
Centre 2	23
Centre 3	10
Centre 4	9
Centre 5	8
Centre 6	7
Centre 7	6
Centre 8	3
Centre 9	2
Centre 10	2
Centre 11	1
Centre 12	1
Total	103

*MR*, magnetic resonance

The main acquisition parameters for the different MRI scanners are reported in Supplementary Table 1.

### Reader agreement

The agreement between readers for each single PI-QUAL score (i.e., PI-QUAL 1 to PI-QUAL 5) was strong, with κw = 0.85 (95% confidence intervals: 0.51 – 1) and percent agreement = 84% (Table 3).

**Table 3:** Overall PI-QUAL scores (n = 103) as assessed by each reader

		Reader 2					Total
		PI-QUAL 1	PI-QUAL 2	PI-QUAL 3	PI-QUAL 4	PI-QUAL 5	
Reader 1	PI-QUAL 1	1	0	0	0	0	1
	PI-QUAL 2	0	14	2	1	0	17
	PI-QUAL 3	0	4	38	1	0	43
	PI-QUAL 4	0	0	8	32	0	40
	PI-QUAL 5	0	0	0	0	2	2
Total		1	18	48	34	2	103

*PI-QUAL*, Prostate Imaging Quality

A strong agreement (κw = 0.82 [95% confidence intervals: 0.68 – 0.96] and percent agreement = 84%) was also observed when the scans were clustered into three groups according to the ability (or not) to rule in and to rule out clinically significant prostate cancer (i.e., PI-QUAL 1-2 vs PI-QUAL 3 vs PI-QUAL 4-5) (Table 4).

**Table 4:** PI-QUAL scores stratified in three different subgroups (n = 103) as assessed by each reader

		Reader 2			Total
		PI-QUAL 1–2	PI-QUAL 3	PI-QUAL 4–5	
Reader 1	PI-QUAL 1–2	15	2	1	18
	PI-QUAL 3	4	38	1	43
	PI-QUAL 4–5	0	8	34	42
Total		19	48	36	103

*PI-QUAL*, Prostate Imaging Quality

The two readers showed disagreement in 16/103 (16%) scans. In detail, 8/16 (50%) scans were scored PI-QUAL 4 by reader 1 and PI-QUAL 3 by reader 2, 1/16 (6%) scan was scored as PI-QUAL 3 by reader 1 and PI-QUAL 4 by reader 2, 4/16 (25%) scans were scored PI-QUAL 3 by reader 1 and PI-QUAL 2 by reader 2, 2/16 (13%) scans were scored as PI-QUAL 2 by reader 1 and PI-QUAL 3 by reader 2, and 1/16 (6%) scan was scored as PI-QUAL 2 by reader 1 and PI-QUAL 4 by reader 2.

As the PI-QUAL scoring sheet (Fig. 1) includes for each MRI sequence a two-step procedure that involves first the application of objective criteria for technical parameters (according to the PI-RADS v. 2.0 guidelines) and then a visual assessment of anatomical structures and image artefacts, we report the scores given by each reader for the 16 scans of disagreement in Table 5.

**Table 5:** Parameters included in the visual assessment of the PI-QUAL scoring sheet in the 16 cases of disagreement

	Reader 1	Reader 2
T2-WI		
Capsule clearly delineated	14	10
Seminal vesicles clearly delineated	14	11
Ejaculatory ducts clearly delineated	11	3
Neurovascular bundles clearly delineated	15	5
Sphincter muscle clearly delineated	14	5
Absence of artefacts	16	13
DWI		
Adequate ADC map	11	5
Absence of artefacts	14	10
DCE		
Capsular vessels clearly delineated	12	5
Vessels in the Alcock’s canal clearly delineated	12	9
Absence of artefacts	15	13

*T2-WI*, T2-weighted imaging; *DWI*, diffusion-weighted imaging; *ADC*, apparent diffusion coefficient; *DCE*, dynamic contrast enhanced

**Fig. 4:**
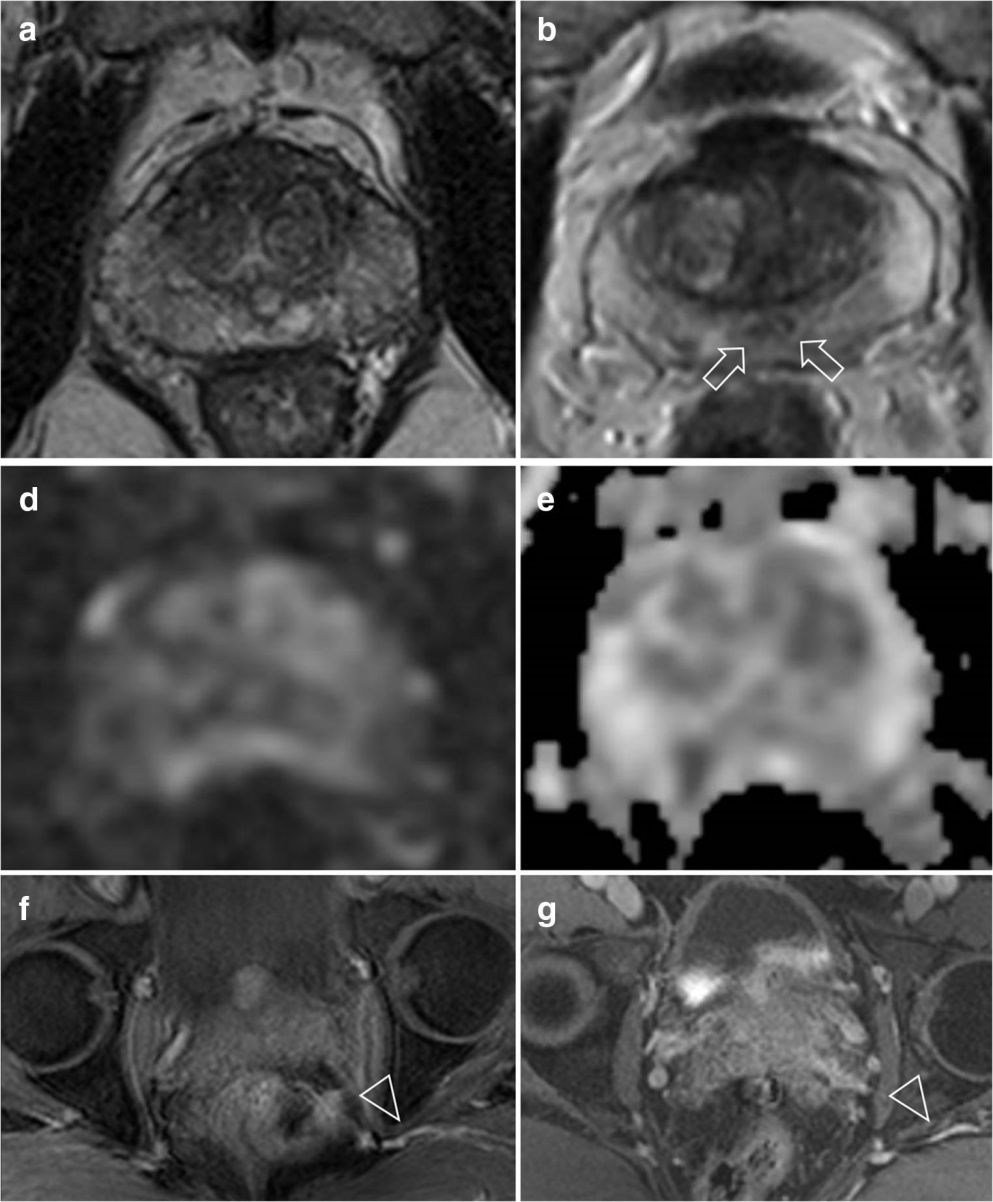
Six examples in which the two readers showed disagreement. *T2-weighted imaging*: in **a** the disagreement pertained to the delineation of the prostatic capsule while in **b** of the ejaculatory ducts (arrows). The final consensus was that the two scans were of suboptimal image quality. *Diffusion-weighted imaging*: in **d** the disagreement pertained to the adequacy of the ADC map (the corresponding high b sequence is provided in **c** for the sake of completeness). The final consensus was that the ADC map was not of adequate diagnostic quality. *Dynamic-contrast enhanced sequences*: the arrowheads in **e** and **f** are indicating the vessels in the Alcock’s (or pudendal) canal in two different patients. One reader scored both scans as of suboptimal quality but after consensus meeting the readers agreed that the Alcock’s (or pudendal) canal was clearly delineated in the two scans

After completion of the study, the two radiologists met and discussed the discordant cases for training and quality purpose. The final scores by consensus were as follows: 6/16 (37.5%) scans were scored as PI-QUAL 2, 4/16 (25%) scans were scored as PI-QUAL 3 and 6/16 (37.5%) scans were scored PI-QUAL 4 (Fig. 4).

As far as field strength is concerned (Supplementary Table 2), 65/83 (78%) and 19/20 (95%) scans had a PI-QUAL score ≥ 3 for 1.5-T and 3-T magnets, respectively. It should be noted that 3-T scanners represented only a small part (19%) of the examinations included in this study.

As far as the diagnostic quality for each single sequence is concerned, the percent agreement between readers was highest for T2-WI (92/103 scans; 89%), followed by DCE sequences (91/103; 88%) and lastly by DWI (80/103; 78%).

**Fig. 5:**
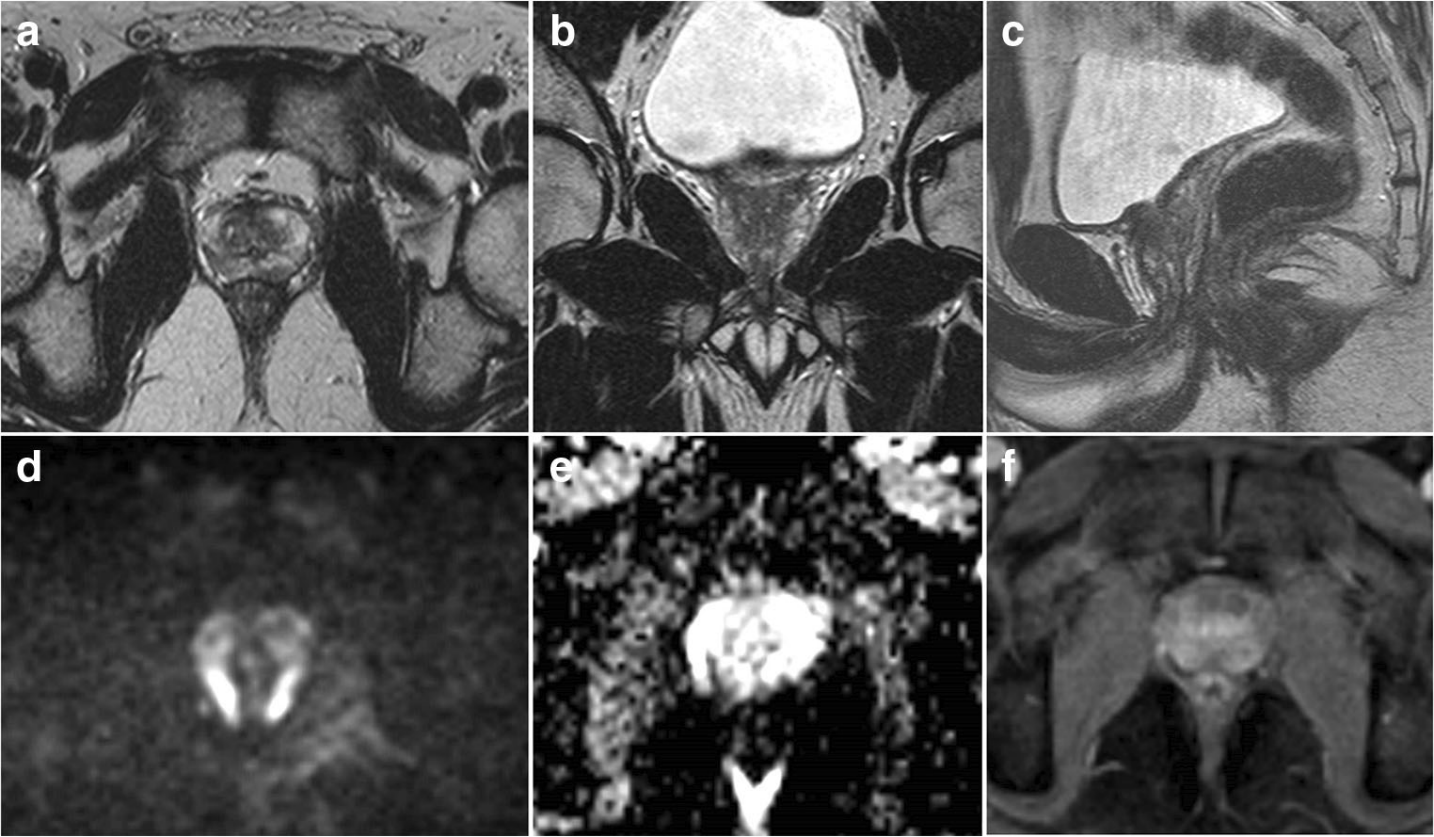
Images of a 50-year-old patient scanned on a 1.5-T MR system with a presenting PSA of 4.7 ng/ml and a prostate volume of 25 cc (PSA density: 0.19 ng/ml/ml), and bilateral Gleason 3 + 4 at biopsy. Axial (**a**), coronal (**b**), and sagittal (**c**) T2-weighted images were judged as the only sequences of acceptable diagnostic quality, as the high *b* sequence (*b* = 1400 s/mm^2^) (**d**) and apparent diffusion coefficient (**e**) map from diffusion-weighted imaging showed artefacts from rectal gas and poor in-plane resolution and dynamic contrast-enhanced (DCE) sequences (**f**) showed suboptimal in-plane resolution and capsular vessels not clearly demarcated. The PI-QUAL score for both readers was 2 (i.e., only one MR sequence is of acceptable diagnostic quality). Final pathology confirmed bilateral Gleason 3 + 4 (pT2c), with an overall tumour volume of 1.7 cc and positive intraprostatic margins on both sides

**Fig. 6:**
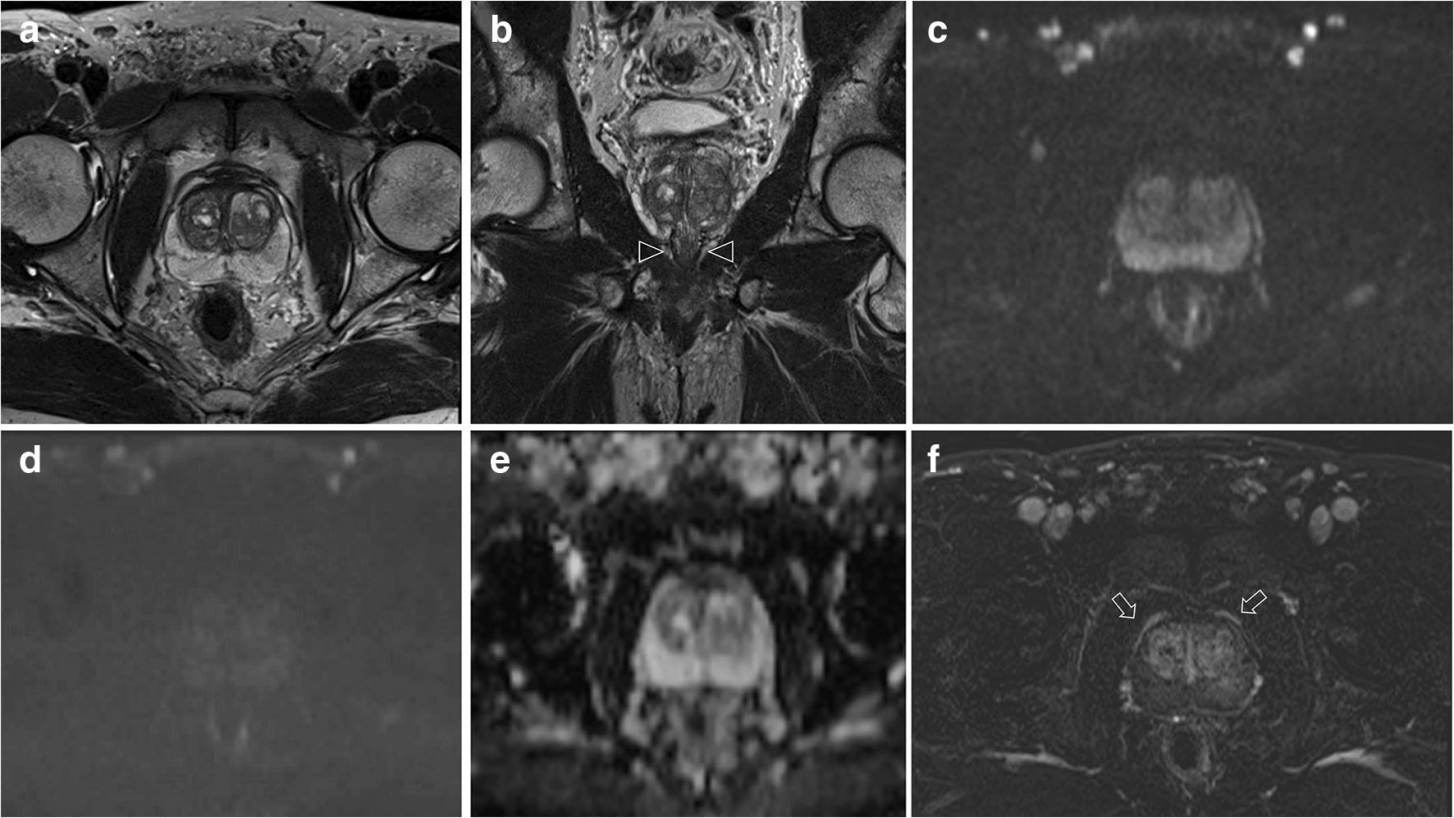
Images in a 50-year-old patient scanned on a 3-T MR system with a presenting PSA of 5 ng/ml and a prostate volume of 80 cc (PSA density: 0.06 ng/ml/ml), and bilateral Gleason 3 + 4 at biopsy. Axial (**a**) and coronal (**b**) T2-weighted images were judged of adequate diagnostic quality, and the arrowheads in (**b**) indicate the internal urethral sphincter clearly demarcated. The *b* = 1000 s/mm^2^ (**c**) and the high *b* sequences (*b* = 1400 s/mm^2^) (**d**) along with the apparent diffusion coefficient (**e**) map from diffusion-weighted imaging were of acceptable diagnostic quality but the in-plane resolution of (**d**) and (**e**) was deemed suboptimal by both readers. The subtracted dynamic contrast-enhanced sequences (**f**) were of adequate diagnostic quality (the arrows indicate the capsular vessels clearly demarcated) but the temporal resolution was 20 s (i.e., above the threshold of 10 s as per PI-RADS v.2.0 guidelines). The PI-QUAL score for both readers was 4 (i.e., two or more mpMRI sequences are independently of diagnostic quality). Final pathology confirmed bilateral organ-confined Gleason 3 + 4 (pT2c), with an overall tumour volume of 0.7 cc

In addition to this, T2-weighted acquisitions were the most compliant with the PI-RADS v. 2.0 technical recommendations across centres and were also the sequences of highest diagnostic quality for both readers in 95/103 (92%) scans, followed by DCE acquisition with 81/103 (79%) scans and lastly by DWI with 79/103 (77%) scans (Figs. 5 and 6).

## Discussion

In our study, we observed strong reproducibility in the assessment of prostate MRI quality using PI-QUAL between two radiologists with high expertise in prostate MRI. The agreement was strong both for each single PI-QUAL score (κw = 0.85 and percent agreement = 84%) and when the PI-QUAL scores were clustered into three groups according to the ability (or not) to rule in and to rule out clinically significant prostate cancer (κw = 0.82 and percent agreement = 84%).

There is currently much interest in standardising high-quality prostate MRI, as this is of paramount importance especially when it comes to MRI-derived targeted biopsies used to detect clinically significant prostate cancer, since a patient with a negative scan and favourable prostate-specific antigen (PSA) kinetics should confidently avoid unnecessary immediate biopsy [13]. However, in order to be able to safely rule in and rule out prostate cancer, images with good spatial resolution, high signal-to-noise ratio and no artefacts are necessary.

The level of reproducibility found in our study compares favourably with that reported for other scoring systems of the prostate [14,15] including PI-RADS [16–21], and similar results have been reported in the arterial hyperenhancement for the diagnosis of hepatocellular carcinoma using a 1-to-5 scoring system [22] and for the Bosniak classification of cystic renal masses [23].

In addition to this, Brembilla and colleagues have recently reported that interobserver studies in prostate MRI research should mirror clinical practice as closely as possible to increase the generalisability of the results [24].

At present, little is known about the effect of imaging quality on interobserver agreement [16] and a recent international consensus meeting has reiterated the importance of studies focusing on reporting MRI quality, as this is critical in studies evaluating prostate MRI performance and reproducibility [3].

The PI-RADS guidelines are well established [25] and, as such, have already been evaluated in studies that have demonstrated their generalisability and reliability. The PI-QUAL score, on the other hand, had been more recently developed and as such it is important to determine the reproducibility of the system [26].

If we have a closer look at Table 5, we can see that the highest levels of disagreement in terms of visual assessment on T2-WI and DCE corresponded to the delineation of specific anatomic landmarks, such as the ejaculatory ducts, the neurovascular bundles and the urethral sphincter muscles on T2-WI along with the vessels on DCE, while higher agreement was observed as far as the prostatic capsule and seminal vesicles on T2-WI are concerned. This could be explained by the fact that some structures (e.g. the ejaculatory ducts and the urethral sphincter muscles) are more difficult to be identified and one of the differences that emerged during the post-scoring consensus meeting was that the senior consultant radiologist in prostate MRI reading used both the axial and coronal acquisitions to judge the visibility of these structures, while the other radiologist used only the axial T2-WI [27]. Also, the visual assessment of DCE images is more difficult compared to T2-WI, as multiple dynamic time-points need to be reviewed without a clear temporal definition for what constitutes early enhancement (e.g. a possible solution could be to use the time point when the adenoma in the transitional zone just starts to enhance, which should occur before any peripheral zone enhancement if the temporal resolution is adequate).

As far as DWI is concerned, the disagreement was substantial when assessing the adequacy of the apparent diffusion coefficient (ADC) map. This is an interesting point, as visual assessment is often used as the primary method to assess the quality of DWI [28] and this could be strengthened by the extrapolation of standardised ADC values from different institutions and MRI scanners or by using built-in phantoms on each scanner.

Interestingly, in the 16 cases reported in Table 5, the two readers had substantial agreement in the evaluation of the absence of artefacts on all sequences, in particular on T2-WI and DCE.

A key feature of our study is its multi-centre setting, which reflects the heterogeneity of prostate MRI conduct across different institutions and is representative of patients undergoing prostate MRI during routine clinical practice, thereby reinforcing the generalisability of our findings. Moreover, both readers interpreted the full examinations on imaging workstations rather than screen captures, ensuring the most accurate assessment for each MRI study.

Our results suggest that the PI-QUAL score can be applied with reasonable consistency by radiologists familiar with prostate MRI on the basis of a clear understanding of the lexicon provided in the scoring sheet [29]. However, it needs to be stressed that only genitourinary radiologists with appropriate training in prostate MRI should assess the quality of the scans [30]. The findings of our study may be also useful for guiding the future iteration of the PI-QUAL score, which should focus on the features that have the greatest objective reproducibility and that should receive more emphasis in the future versions.

Also, a more objective definition of some less reproducible features such as the ‘reader-estimated’ criteria (e.g. what constitutes ‘adequacy’ of the ADC map) will need to be addressed in the next version of PI-QUAL.

Our study has some limitations. Firstly, both readers assessing the PI-QUAL score worked at the same centre and the junior consultant radiologist had been previously trained by the senior consultant radiologist; thus, they may have had the tendency to approach cases similarly. It should be also noted that the PI-QUAL score has been only recently published [5] and there is currently no literature on its application in a clinical setting.

Another limitation of the present study is that we did not investigate the correlation of PI-QUAL with radical prostatectomy. Although we know that the use of PI-QUAL has clinical implications (i.e., ruling in and ruling out clinically significant prostate cancer), the aim of this specific study was to test the interobserver reproducibility of the PI-QUAL score and not to evaluate the correlation between image quality, lesion conspicuity on mpMRI and histology. Though we did not test the inter-reader variability in ruling in and ruling out clinically significant prostate cancer, we can anticipate that other work is already underway to investigate PI-QUAL score and diagnostic accuracy with pathology as reference standard. It should be also noted that the population included in this study consisted of patients who were candidates for radical prostatectomy, so there could have been a bias towards those studies with MR-visible lesions and higher PI-RADS scores.

As previously stated in the original publication [5], the PI-QUAL score will be refined in the future (e.g. an international group is currently working on the next version of PI-QUAL in order to see if this scoring system should be still based on a 5-point scale or simplified into a 3-point scale) and we believe that the findings of our study may be useful for guiding the future iteration of this scoring system. Thus, it is envisaged that the interobserver agreement will continue to improve over time with the new iterations of the PI-QUAL score.

In conclusion, the findings of our study support the reliability of the PI-QUAL score for the assessment of prostate MRI quality. However, it is anticipated that the scoring system will need to undergo further refinements.

## Supplementary Information

ESM 1 (DOCX 23 kb)

## Abbreviations

ADC: Apparent diffusion coefficient
DCE: Dynamic contrast enhanced
DWI: Diffusion-weighted imaging
mpMRI: Multiparametric magnetic resonance imaging
PACS: Picture Archiving and Communication System
PI-QUAL: Prostate Imaging Quality
PI-RADS: Prostate Imaging Reporting and Data System
PSA: Prostate-specific antigen
T2-WI: T2-weighted imaging

## Declarations

### Guarantor

The scientific guarantor of this publication is Francesco Giganti.

### Statistics and biometry

No complex statistical methods were necessary for this paper.

### Methodology

• retrospective

• randomised controlled trial

• multi-centre study
