# Supplementary material for: Inter-reader agreement of the PI-QUAL score for prostate MRI quality in the NeuroSAFE PROOF trial
Source: Eur Radiol. 2021 Jul 29;32(2):879–89. doi: 10.1007/s00330-021-08169-1 (PMC8794934; doi:10.1007/s00330-021-08169-1)
Supplement: Supplementary file 1 — (DOCX 23 kb) [file 330_2021_8169_MOESM1_ESM.docx]

|  |  | Centre 1 | Centre 2 | Centre 3 | Centre 4 | Centre 5 | Centre 6 | Centre 7 | Centre 8 | Centre 9 | Centre 10 | Centre 11 | Centre 12 |
| --- | --- | --- | --- | --- | --- | --- | --- | --- | --- | --- | --- | --- | --- |
| T2-WI |  | | | | | | | | | | | | |
|  | Axial plane | Yes | Yes | Yes | Yes | Yes | Yes | Yes | Yes | Yes | Yes | Yes | Yes |
|  | Sagittal / coronal plane | Yes | Yes | Yes | Yes | Yes | Yes | Yes | Yes | Yes | Yes | Yes | Yes |
|  | Field of view (cm) | 14 x 18 | 17 x 17 | 17 x 17 | 19 x 19 | 18 x 14 | 22 x 22 | 17 x 14 | 16 x 14 | 17 x 18 | 19 x 19 | 20 x 20 | 18 x 19 |
|  | Adequate in-plane resolution | Yes | Yes | Yes | Yes | No | Yes | No | Yes | Yes | Yes | No | Yes |
|  | Slice thickness | 3 | 3 | 3 | 3 | 3 | 4 | 3 | 3.5 | 3 | 3.5 | 3 | 3 |
|  | Z-axis correctly positioned | Yes | Yes | Yes | Yes | Yes | Yes | Yes | Yes | Yes | Yes | Yes | Yes |
| DWI |  | | | | | | | | | | | | |
|  | Axial plane matching T2-WI | Yes | Yes | Yes | Yes | Yes | Yes | Yes | Yes | Yes | Yes | Yes | Yes |
|  | Field of view (cm) | 18 x 18 | 22 x 23 | 27 x 36 | 18 x 21 | 14 x 18 | 21 x 37 | 15 x 20 | 24 x 28 | 20 x 21 | 22 x 25 | 16 x 19 | 12 x 23 |
|  | Adequate in-plane resolution | Yes | No | No | Yes | Yes | Yes | No | Yes | No | Yes | No | Yes |
|  | Slice thickness | 5 | 3 | 6 | 5 | 5 | 3 | 6 | 5 | 6 | 5 | 4 | 5 |
|  | Multiple b values | Yes | Yes | Yes | Yes | Yes | Yes | Yes | Yes | Yes | Yes | Yes | Yes |
|  | High b value (s/mm^2^) | 2,000 | 1,200 | No | 2,000 | 2,000 | 1,200 | No | No | 1,200 | No | 1,400 | 1,600 |
| DCE |  | | | | | | | | | | | | |
|  | Axial plane matching T2-WI | Yes | Yes | Yes | Yes | Yes | Yes | Yes | Yes | Yes | Yes | Yes | Yes |
|  | Field of view (cm) | 22 x 24 | 20 x 20 | 20 x 33 | 21 x 23 | 22 x 25 | 24 x 26 | 23 x 27 | 19 x 23 | 18 x 17 | 21 x 25 | 22 x 25 | 19 x 23 |
|  | Adequate in-plane resolution | Yes | Yes | Yes | Yes | Yes | Yes | No | No | No | Yes | No | No |
|  | Slice thickness | 3 | 2 | 2 | 3 | 3 | 6 | 4 | 3 | 4 | 3.5 | 3.5 | 3.4 |
|  | Pre-contrast T1-WI | Yes | Yes | Yes | Yes | Yes | Yes | Yes | Yes | Yes | Yes | Yes | Yes |
|  | Fat suppression/subtraction | Yes | Yes | Yes | Yes | Yes | Yes | Yes | Yes | Yes | Yes | Yes | Yes |
|  | Temporal resolution (sec) | 15 | 19 | 13 | 18 | 20 | 16 | 11 | 27 | 14 | 12 | 15 | 16 |
|  | Total observation rate (min) | 5:27 | 2:40 | 4:51 | 3:53 | 5:03 | 4:29 | 1:46 | 3:01 | 3:08 | 6:40 | 3:12 | 5:08 |

**Supplementary Table 1** – Main acquisition parameters of the MR scanners included in the study.

Legend – T2-WI: T2-weighted imaging; DWI: diffusion-weighted imaging; DCE: dynamic-contrast enhanced; T1-WI: T1-weighted imaging

**Supplementary Table 2** – PI-QUAL scores stratified by field strength (1.5T vs 3T)

|  | 1.5 T | 3 T |  |
| --- | --- | --- | --- |
| PI-QUAL 1 | 1 | - | 1 |
| PI-QUAL 2 | 17 | 1 | 18 |
| PI-QUAL 3 | 40 | 8 | 48 |
| PI-QUAL 4 | 24 | 10 | 34 |
| PI-QUAL 5 | 1 | 1 | 2 |
| Total | 83 | 20 | 103 |

Legend - PI-QUAL: Prostate Imaging Quality
